# The effect of silencing immunity related genes on longevity in a naturally occurring *Anopheles arabiensis* mosquito population from southwest Ethiopia

**DOI:** 10.1186/s13071-019-3414-y

**Published:** 2019-04-16

**Authors:** Serkadis Debalke, Tibebu Habtewold, Luc Duchateau, George K. Christophides

**Affiliations:** 1 Department of Medical Laboratory Science & Pathology, Jimma University, Jimma, Ethiopia; 2 Biometrics Research Group, Ghent University, Ghent, Belgium; 3 Department of Life Sciences, Imperial College London, London, UK

**Keywords:** *Anopheles arabiensis*, Longevity, Gene silencing, Microbiota, Gut homeostasis

## Abstract

**Background:**

Vector control remains the most important tool to prevent malaria transmission. However, it is now severely constrained by the appearance of physiological and behavioral insecticide resistance. Therefore, the development of new vector control tools is warranted. Such tools could include immunization of blood hosts of vector mosquitoes with mosquito proteins involved in midgut homeostasis (anti-mosquito vaccines) or genetic engineering of mosquitoes that can drive population-wide knockout of genes producing such proteins to reduce mosquito lifespan and malaria transmission probability.

**Methods:**

To achieve this, candidate genes related to midgut homeostasis regulation need to be assessed for their effect on mosquito survival. Here, different such candidate genes were silenced through dsRNA injection in the naturally occurring *Anopheles arabiensis* mosquitoes and the effect on mosquito survival was evaluated.

**Results:**

Significantly higher mortality rates were observed in the mosquitoes silenced for *FN3D1* (AARA003032), *FN3D3* (AARA007751) and *GPRGr9* (AARA003963) genes as compared to the control group injected with dsRNA against a non-related bacterial gene (*LacZ*). This observed difference in mortality rate between the candidate genes and the control disappeared when gene-silenced mosquitoes were treated with antibiotic mixtures, suggesting that gut microbiota play a key role in the observed reduction of mosquito survival.

**Conclusions:**

We demonstrated that interference with the expression of the *FN3D1*, *FN3D3* or *GPRGr9* genes causes a significant reduction of the longevity of *An. arabiensis* mosquito in the wild.

**Electronic supplementary material:**

The online version of this article (10.1186/s13071-019-3414-y) contains supplementary material, which is available to authorized users.

## Background

Sub-Saharan Africa hosts some of the most efficient malaria vectors, *An. gambiae*, *An. arabiensis* and *An. funestus*, and carries the heaviest malaria burden worldwide. A substantial reduction in malaria related cases and deaths have been recorded in the past decade [1]. This progress is largely attributable to the scaling-up of vector control interventions, such as long-lasting insecticide-treated nets (LLINs) and indoor residual spraying (IRS), as well as improved diagnostics and effective treatment using artemisinin-based combination therapies [2].

LLINs and IRS impact malaria transmission largely by reducing the daily survival rate of mosquitoes that are mostly active at night and display strong endophagic (seeking blood meals indoors) and endophilic (rest indoors following a blood meal) behavior [3, 4]. These tools have been more efficient for the endophagic/endophilic *An. gambiae* and *An. funestus* mosquitoes compared to the exophagic/exophilic *An. arabiensis* mosquito [5–9]. In addition, field studies have reported evidence of behavioral adaptation of *An*. *arabiensis* to the LLINs and/or ITNs as mosquitoes feed earlier and more frequently outdoors, and rest more frequently outdoors [7, 10, 11].

Due at least in part to this difference in vector control efficacy, a marked shift in vector composition has been observed. In east African countries where these vectors coexist, *An. arabiensis* is gradually replacing *An. gambiae* and *An. funestus*, e.g. in Kenya [12–14] and Tanzania [7, 15]. Although *An. arabiensis* is known to be a less efficient vector compared to *An. gambiae* and *An. funestus*, the inherent resilience of the mosquito to LLINs and IRS has been linked to reports of resurgence or stagnation in rates of malaria cases and deaths in African countries [14]. Therefore, there is a need to develop new complementary vector control technologies targeting vectors that are resilient to the current vector control tools.

Malaria transmission blocking vaccines and mosquito population replacement *via* genetic modification have recently become attractive technologies to complement the existing vector control interventions [16–21]. Another novel approach is to reduce the mosquito lifespan by introducing a lethal gene or a pathogen in the mosquito population [22, 23]. Along the same lines, the mosquito lifespan could be reduced by immunizing primary mosquito blood hosts (i.e. humans and domestic bovids) with mosquito proteins involved in the midgut homeostasis. For this approach, candidate genes need to be found, which is the topic of this paper. Different *An. arabiensis* genes related to midgut homeostasis were screened for their potential to reduce mosquito longevity. Our work was prompted by data showing that shortly after a blood meal the number of microbiota in the mosquito midgut increases drastically, up to 1000 times [24–26], which in normal circumstances triggers immune reactions that soon reduce the microbiota number to the basal level [27–31]. We hypothesized that by compromising the immune system the mosquito would no longer be able to control the microbiota in the gut, which would lead to a shorter lifespan. Previous studies have demonstrated in a laboratory colony of *An. gambiae*, that when genes encoding putative bacterial receptors such as *PGRPLC*, type III fibronectin domain proteins (FN3Ds including *FN3D1*, *FN3D2*, *FN3D3*) and the gustatory receptor *GPRGr9* were silenced, gut homeostasis was disrupted [28, 32]. Here, we investigated the effect of silencing some of these genes by RNA interference on longevity in naturally occurring *An. arabiensis* populations.

## Methods

### Mosquitoes

Adult *An. arabiensis* mosquitoes were reared from larvae and pupae collected from natural breeding sites around Jimma, south-west Ethiopia (07°40′00″N, 36°50′00″E). Larvae and/or pupae were collected using a 350 ml mosquito dipper following the standard larvae collection procedure [33, 34]. The collected larvae and pupae were transported to an on-site mud-house where the pupae were separated from the larvae and transferred into a 10 ml beaker with water and kept in a mosquito cage with a dimension of 24.5 × 24.5 × 24.5 cm^3^ (Bugdorm-41515; Watkins & Doncaster, Leominster, UK) until emergence. The remaining larvae were transferred to a plastic tray containing water obtained from their natural habitat and fed on yeast and tropical fish food. The water was changed every 2 days and pupae were collected daily and transferred to the adult cage. Emerged adult mosquitoes were maintained on 10% sugar solution. Adult females of zero to two day old were transported to the experimental insectary at Jimma University for double stranded RNA (dsRNA) injection.

### Gene silencing

Total RNA was extracted from ten field-collected and laboratory reared *An. arabiensis* mosquitoes using TRIzol (Invitrogen, Inchinnan, UK) and cleaned with Turbo DNase I (Ambion, Huntington, UK). Complementary DNA (cDNA) was synthesized by reverse transcribing 1 μg of the total RNA using a Prime-Script™ 1^st^-strand cDNA Synthesis Kit (TaKaRa, Kusatsu, Shangai). Fragments of the five target genes were amplified by PCR using specific gene primers tailed with the short T7 promoter sequence TAA TAC GAC TCA CTA TAG G and the cDNA as a template. The targeted genes were *FN3D1* (AARA003032), *FN3D2* (AARA007751), *FN3D3* (AARA007751), *GPRGR9* (AARA003963) and *PGRPLC3* (AARA002982). PCR fragment for the *LacZ* gene that served as a control was synthesized using a plasmid template containing the *LacZ* gene (for full primer sequences see Additional file 1: Table S1). DsRNA was synthesised from purified PCR products using the TranscriptAid T7 High Yield Transcription Kit (Thermo Fisher Scientific, Waltham, USA). The dsRNA was then purified using an RNeasy Mini Kit (Qiagen, Manchester, UK), following the manufacturer’s protocol. The concentration of dsRNA was determined spectrophotometrically by a Nanodrop 1000 spectrometer (Thermo Fisher Scientific, Wilmington, USA) at 260 nm and adjusted to 3 μg/μl using ultra-pure water. Gel electrophoresis (1% TBE agarose) was performed on a sub-sample of the PCR products to confirm that products of the expected size were detected for each gene. Zero- to two-days-old *An. arabiensis* mosquitoes were injected with 69 nl of dsRNA specific to a target gene or the *LacZ* control gene following a published RNA interference technique [35].

Gene silencing efficiency was measured for each of the 5 silenced genes using qrtPCR. Quantification of transcript abundance was performed on cDNA synthesized from total RNA extracted from mosquitoes injected with dsRNA 3 days earlier and maintained on 10% sugar solution. Fast SYBR^®^ Green Master Mix Real-Time PCR Master Mix (Applied Biosystems, Warrington, UK) was used in the PCR reaction and amplification was detected by a 7500 Fast Real-Time PCR system (Applied Biosystems). Each target gene was quantified in duplicate. The *AgS7* gene was used as an internal control. Primer sequences are given in Additional file 1: Table S1.

### Monitoring of mosquito survival

The dsRNA injected mosquitoes were put into their respectively labelled cups. For each gene between 20 and 30 mosquitoes were injected per replicate. Cups with the mosquitoes were placed inside a 2 × 1 × 0.75 m^3^ (length × width × height) microclimate regulatory box constructed from chipboard. It has a window of 25 cm^2^ on each side and the top cover, and was covered with metal mesh to allow airflow (Additional file 2: Figure S1). The box was placed in a typical rural house. To prevent ant attacks, the box was placed on a 50 cm raised stand that was dipped halfway into water. It was lined with sawdust, 30 cm deep, which was sprinkled daily with water and kept closed. The box maintained a humidity of 60–70% RH and temperature of 25–28 °C throughout the whole study period, hence providing a microclimatic condition that resembled the natural resting habitat of *An. arabiensis* mosquitoes. The mosquitoes were offered a 10% sugar solution daily and a blood meal every 4 days by direct-feeding on a goat. Survival was monitored daily for 25 days, starting 24 h post-injection. Six independent replicates of mosquito injection were performed per gene.

### Midgut microbiota analysis

A separate experiment was performed to determine microbiota load in both blood-fed and sugar-fed mosquitoes injected with the specified dsRNA. For each gene, between 20 and 30 mosquitoes were injected and transferred to their corresponding labelled cups. The mosquitoes were then kept in a microclimate regulatory box. On day 4 post-injection, the mosquitoes were either fed on blood or kept on a sugar meal. Twenty-four hours post-feeding, 5 blood-fed and 5 sugar-fed mosquitoes were sampled and their midguts were dissected. Individual midguts were homogenized in 100 µl of 4% paraformaldehyde (PFA) in phosphate-buffered saline (PBS). The number of bacteria was quantified using flow cytometry on the 5 pooled midgut samples per replicate [35]. A total of 3 replicates of dsRNA injections were carried out per gene.

### Antibiotic treatment

We tested the effect of reducing/eliminating the midgut microbiota load on the survival of the gene knocked down mosquitoes. For this purpose, dsRNA-injected mosquitoes were placed in 6 different cups, each cup containing 20–30 mosquitoes. The cups were kept in the microclimate regulatory box. On the day of dsRNA injection, the mosquitoes were given a cotton ball soaked in an antibiotic cocktail of streptomycin and norfloxacin, both at a dose rate of 10 μg/ml in a 10% sugar solution. On day 4 post-injection, the mosquitoes were blood-fed on a goat. The cotton balls were re-soaked with the antibiotic cocktail every fourth day for a period of 12 days (on days 4, 8 and 12). The antibiotics/sugar feed was carried out in the morning for about 1 h, and then cotton ball was removed from the cups for the next 5 h to starve the mosquitoes. The starved mosquitoes were offered a blood meal and the cotton ball was replaced on the cups after the feed. Mosquito survival was monitored daily for 25 days starting from 24 h post-injection. For this experiment 5 replicates of dsRNA injection were performed per gene.

### Data analysis

Data analysis was performed using the statistical software package R v.3.3.2. For the gene silencing efficiency test, the relative expression of mRNA was calculated. The standard curve method was used for real-time qPCR quantification analysis. For each test sample, the PCR cycle number at which the fluorescent intensity of the reaction curve intersects the threshold line, i.e. level of detection or the point at which a reaction reaches a fluorescent intensity above background levels, known as threshold crossing values (Ct-values), was determined for the target and reference gene. Additionally, a standard curve was generated for both the target gene and the reference gene in each assay run using serial dilution of same template. Then, for each sample, the Ct-value of the target or reference gene was standardized using corresponding a standard curve and then the expression level of the target gene was normalized to the reference gene (AgS7 gene). The relative expression of mRNA of a target gene was compared between mosquitoes for which the target gene was silenced, and mosquitoes injected with control ds*LacZ* by a paired t-test using the replicate as a block factor. Gene silencing efficiency was expressed as the ratio of the relative expression of the target gene in the ds*LacZ* injected mosquitoes and the target gene silenced mosquitoes. Survival of the target gene and *LacZ*ds RNA-injected mosquitoes was depicted by Kaplan-Meier survival curves. The effect of silencing the different target genes on survival was modeled by the Cox proportional hazards frailty model, with replicate used as frailty term [36]. The hazard ratio of a target gene over the control ds*LacZ* injection was used as summary statistic, together with the median time to death for the different gene silenced mosquitoes. To investigate the effect of the antibiotics cocktail, the same Cox proportional hazards model was fitted using the hazard ratio again as summary statistic. The bacterial counts were first log-transformed and then compared by a mixed model with replicate as random effect, and treatment, feed and the two-way interaction as fixed effects factor. The F-test was used to compare the silencing of the different target genes and the injection of control ds*LacZ*. The ratios of bacterial counts in the control ds*LacZ* injections and the target gene silencing were used as summary statistics.

## Results

### Effect of gene silencing on survival

**Fig. 1:**
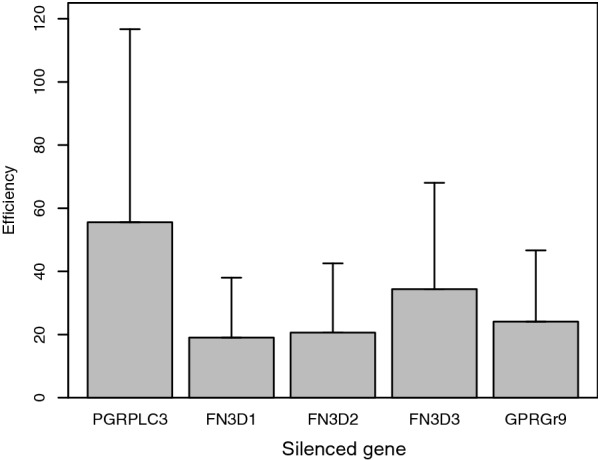
The average reduction (vertical line is standard error) in expression level of mRNA for the specific knocked-down gene mosquitoes as compared to *LacZ* knocked-down mosquitoes (*n* = 3)

A significant reduction in expression level of mRNA compared to the ds*LacZ*-injected mosquitoes was achieved for the targeted candidate genes *FN3D1* (*t* = -8.36, *df* = 2, *P* = 0.007), *FN3D2* (*t *= -7.09, *df* = 2, *P* = 0.010), *FN3D3* (*t* = -3.82, *df* = 2, *P* = 0.031) and *GPRGr9* (*t* = -6.60, *df* = 2, *P* = 0.011) but not for *PGRPLC3* (*t* = -1.42, *df* = 2, *P* = 0.145). The data are presented in Fig. 1.

**Fig. 2:**
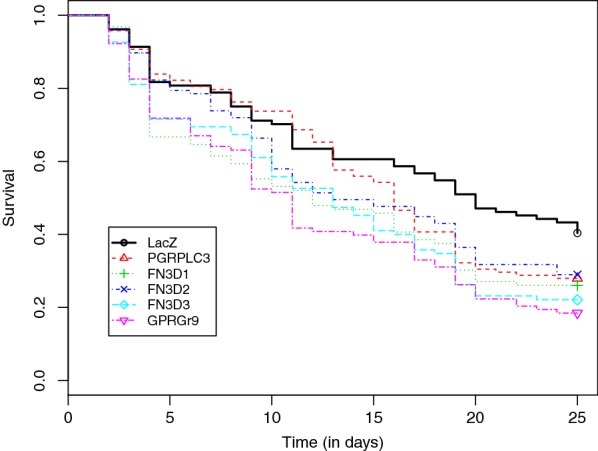
Kaplan-Meier curves depicting the survival rate as a function of time for gene-silenced mosquitoes. In the experiment, five test and one control genes were considered. The Kaplan-Meier curve for each gene is based on six replicates each consisting of 20–30 mosquitoes

Mosquito survival following silencing of the target genes compared to the control ds*LacZ* injected mosquitoes is depicted as a function of time for the each of the above genes in Fig. 2. Significantly higher mortality rates were observed for the *FN3D1*, *FN3D3* and *GPRGr9* knocked-down mosquitoes as compared to the control group, but not for the *FN3D2* and *GRPLC3* knocked-down mosquitoes (Table 1).

**Table 1:** Effect of gene silencing on mosquito survival. The second column presents the hazard ratio (HR) of dying between a gene knockdown and ds*LacZ* control. The third column presents the hazard ratio of dying between a gene knockdown and ds*LacZ* control when mosquitoes are treated in parallel with antibiotics

Gene	Hazard ratio (95% CI; *P*-value)	
	Without antibiotics	With antibiotics
*LacZ*	1.00	1.00
*FN3D1*	1.64 (1.17–2.30; *P* = 0.004)	1.02 (0.75–1.37; *P* = 0.91)
*FN3D2*	1.40 (1.00–1.95; *P* = 0.050)	1.17 (0.86–1.58; *P* = 0.32)
*FN3D3*	1.79 (1.28–2.50; *P* < 0.001)	0.90 (0.67–1.21; *P* = 0.50)
*GPRGr9*	2.00 (1.45–2.76; *P* < 0.001)	0.90 (0.67–1.21; *P* = 0.47)
*PGRPLC3*	1.35 (0.97–1.87; *P* = 0.072)	0.89 (0.66–1.21; *P* = 0.46)

A marked reduction in average time to death was observed in mosquito groups where target genes were silenced compared to ds*LacZ*-injected controls, e.g. for GRPGr9 silenced mosquitoes the median time to death was almost halved from 20 (LacZ) to 11 days (Table 2).

**Table 2:** Median time to death in *An. arabiensis* mosquitoes when silenced with genes through injection of gene-specific dsRNA

Gene	Median time to death (days) (95% CI)
*LacZ*	20 (16– +∞)^a^
*FN3D1*	12 (9–17)
*FN3D2*	13 (10–19)
*FN3D3*	13 (10–17)
*GPRGr9*	11 (9–15)
*PGRPLC3*	16 (13–19)

^a^The upper limit is infinity because some mosquitoes were right censored in this treatment group

### Effect of gene silencing on the midgut bacterial count

The global analysis demonstrated that there was no overall significant difference between the target gene silenced and the ds*LacZ*-injected mosquitoes with respect to bacterial count (*F*_(5,24)_ = 1.148, *P* = 0.363). However, there was an overall effect of the feed source (*F*_(5,24)_ = 20.287, *P* < 0.001), i.e. blood-feeding *versus* sugar-feeding, whereas the interaction between the factors was not significant (*F*_(5,24)_ = 1.902, *P* = 0.131). The ratio of bacteria count in blood-fed mosquitoes as compared to sugar-fed mosquitoes was 2.13 (95% CI: 1.49–3.06).

When considering the blood-fed mosquitoes alone, no statistically significant differences in the bacterial count of the target gene silenced and the ds*LacZ*-injected mosquitoes were observed. The ratio of midgut bacterial count of target gene silenced to the ds*LacZ*-injected mosquitoes is presented in Table 3; the ratios for all target gene silenced mosquitoes compared to the control groups (*LacZ* injected mosquitoes) were above 1, with the highest value for *FN3D1* equal to 2.66 (95% CI: 0.94–7.57) but not significantly different from 1 (*P* = 0.085).

**Table 3:** Bacterial count in *An. arabiensis* mosquitoes silenced with genes involved in midgut homeostasis using by microinjection of gene-specific dsRNA

Gene	Bacterial count ratio (95% CI; *P*-value)
*LacZ*	1.00
*FN3D1*	2.66 (0.94–7.57; *P* = 0.085)
*FN3D2*	1.15 (0.40–3.27; *P* = 0.793)
*FN3D3*	1.36 (0.48–3.85; *P* = 0.570)
*GPRGr9*	2.30 (0.81–6.54; *P* = 0.136)
*PGRPLC3*	1.08 (0.38–3.07; *P* = 0.886)

Treatment of mosquitoes with an antibiotics cocktail eliminated the gene silencing effect on survival for all 5 target genes. No statistically significant differences were noted in terms of mortality between the target genes silenced and the ds*LacZ*-injected mosquitoes (Table 1).

We also observed that for best performing genes, FN3D1 and FN3D3, dsRNA treatment had no effect on mosquito fecundity (number of eggs laid/female) or fertility (egg hatchability) of the mosquitoes (Additional file 3: Table S2).

## Discussion

The scale-up of vector control interventions in conjunction with early patient diagnosis and therapy has resulted in a substantial reduction in malaria-related cases and deaths since 2000. However, the newest data suggest that this progress is progressively coming to a halt or, in fact, being reversed in some countries, indicating that the current malaria intervention tools and strategies may have reached their maximum capacity [1]. This highlights the urgency of developing new tools to complement LLINs to achieve the malaria elimination agenda in Africa, especially against the exophagic and exophilic *An. arabiensis* mosquito. A viable option against this opportunistic feeder species is to challenge different blood hosts with whole protein or antigenic peptide generated from mosquito itself (i.e. mass vaccination). The resulting host antibody is ingested by the mosquito during blood-feeding on an immunised host. This anti-mosquito antibody can be designed to target mosquito molecules involved in midgut homeostasis to reduce the longevity of the mosquito to such an extent that insufficient time remains to transmit the *Plasmodium* parasite.

In order for a mosquito to transmit a pathogen, it must acquire a blood meal from an infected person, support pathogen replication, dissemination to the salivary glands and take a subsequent blood meal from a susceptible host; this period is commonly called extrinsic incubation period (EIP). The EIP ranges from 10 to 14 days (2 to 6 gonotrophic cycles in areas of high malaria transmission) and a female mosquito must survive longer than the EIP to transmit the parasite [37], hence a very small fraction of mosquitoes (< 10%) can survive long enough to transmit the pathogen. This underlines the fact that mosquito longevity is a critical factor affecting malaria transmission and provides a basis for guiding vector control strategies [38, 39]. A small reduction in the mean mosquito survival period would have a significant impact on the disease transmission [40–43].

In the present study, we assessed five midgut-expressed genes *FN3D1*, *FN3D2*, *FN3D3*, *GPRGr9* and *PGRPLC* for their potential as lifespan-limiting targets in a wild *An. arabiensis* population. Previous studies with *An. gambiae* under laboratory settings have demonstrated that the above genes regulate midgut microbiota [28, 32]. Unlike these studies, our work focused on the effect of those genes on the survival of field-caught *An. arabiensis*. Our experimental mosquitoes were captured as larvae or pupae and maintained in an environment simulating the mosquito natural resting habitat. They were also allowed to blood-feed on a goat every fourth day, mimicking their natural habits. We have demonstrated that in these semi-natural conditions, the reduction of *FN3D1*, *FN3D3* or *GPRGr9* expression significantly reduces the longevity of the *An. arabiensis* mosquitoes to an average of 12, 13 and 11 days, respectively, compared to a 20-day average longevity of control mosquitoes.

The observed effect on the mosquito survival is probably linked to the disruption of the mosquito midgut homeostasis as the observed effect of gene silencing (particularly of *FN3D1*, *FN3D3* and *GPRGr9*) on their survival was no longer seen when mosquitoes were treated with an antibiotic cocktail to eliminate their gut microbiota. Our hypothesis that the reduced survival is due to the inability of mosquitoes to control their gut microbiota is also supported by other studies [44, 45]. Furthermore, it has been previously demonstrated that *FN3Ds* and *GPRGr9* have a specific effect on microbiota of the family *Enterobacteriaceae* [32].

Our results demonstrate that interfering with the expression and/or function of these genes reduces the mosquito lifespan which in turn will significantly reduce malaria transmission. A permanent gene inactivation/knock-out can be achieved in various ways. A new and most promising tool is the recently developed CRISPR/Cas9-based genome editing methodology and gene-drive systems for *Anopheles* mosquitoes [21, 46, 47]. Recently a CRISPR/Cas9-induced somatic gene disruption technique has been established in *An. gambiae* [47, 48]. Such a knock-out line can be crossed with a germline-*Cas9* strain as described in to generate a germ-line gene-knockout line which might be released to introgress the life-shortening trait into the wild malaria vector population [49]. A second approach could be through the immunization of the blood-providing host, whether human or domestic animals, with molecules derived from target vector proteins that play a role in midgut homeostasis. The purified form of such molecules (i.e. antigens) can be inoculated into the vertebrate host to induce host immune reaction, and ultimately produce specific antibodies. Mosquitoes feeding on the immunized hosts would then ingest antibodies that neutralize the function of the protein leading to disrupted midgut homeostasis and shortened lifespan. It has been previously demonstrated that *An. gambiae*-derived anti-midgut monoclonal antibodies significantly reduce vector survivorship [50]. This approach is particularly attractive for zoophilic mosquitoes such as *An. arabiensis.* Mosquitoes that have taken an infectious blood meal will typically take three to four additional blood meals before the completion of a sporogonic period as they normally blood-feed every 2–3 days. This could ensure repeated ingestion of anti-mosquito antibodies with consequential disruption of gut bacterial homeostasis to ultimately induce reduced lifespan. This technology can also impact secondary malaria vectors including *Anopheles rivolorum*, *Anopheles pharoensis*, *Anopheles coustani*, *Anopheles ziemanni* and *Anopheles squamosus* that are responsible for about 5% of total malaria transmission in Africa and are often highly zoophilic [51].

As the anti-mosquito vaccine technologies are expected to only reduce the long-term survival of vectors of the mosquitoes, female mosquitoes will complete some of their gonotrophic cycle. Thus, the technologies are expected to have minimum selection pressure inducing resistance [52].

This approach has been successfully used for an anti-tick vaccine targeting the midgut antigens Bm86 and an anti-tick and anti-mosquito vaccine targeting the subolesin/akirin (SUB/AKR) antigens [53–56]. The functional model for the SUB/AKR vaccine involves the nuclear factor-kappa B (NF-kB) of vector insects to inhibit the Immunodeficiency (Imd) pathway, which is important for regulation of the gut microbiota [57, 58].

A major challenge with such protein antigens is that they can result in autoimmunity due to molecular mimicry as such molecules can possess ‘mimotopes’ that are peptides mimicking the antigenic conformation structures that are recognized by the paratope antibody, leading to autoimmunity [59].

In the present case, amino acid sequence analysis of the *An. arabiensis* FN3D1 and FN3D3 genes has shown the presence of 25–37% sequence identity with proteins of some human genes such as the protein tyrosine phosphatase receptor type F gene. This level of homology is often referred as the ‘twilight zone’ where the structural similarity between the target mosquito genes and the human genes cannot be ruled out, suggesting a potential risk of induction of autoimmune diseases in individuals upon immunization with the mosquito genes [60]. For instance, an analysis involving over a million sequences with known structures showed that at the top cut-off of the twilight zone, about 90% of protein pairs were structurally homologous, but the level of homology reduced drastically (to < 10%) when the sequence similarity between protein pairs was below 25% [59]. On the other hand, both mosquito genes have no significant sequence similarity in the domestic Bovidae, hence induction of an autoimmune reaction in the vaccinated animals is negligible and the protein molecules can be used to develop vaccines to immunize the animals with minimum risk.

## Conclusions

Eliminating the expression of the midgut proteins *FN3D1*, *FN3D3* or *GPRGr9* significantly reduces the lifespan of naturally occurring *An. arabiensis* mosquitoes reared in field conditions. The effect is probably caused by disruption of the mosquito midgut homeostasis through interference with the midgut microbiota, eventually hampering the mosquito immuno-metabolic functions. Therefore, these proteins can be good targets of mosquito life-shortening interventions, such as anti-mosquito vaccines or mosquito genetic modification, resulting in mosquitoes that can survive long enough to complete a gonotrophic cycle but not long enough to transmit malaria parasites to a new host. Thus, the technologies are expected to have minimum selection pressure inducing resistance.

## Additional files

**Additional file 1: Table S1.** List of primers used in the dsRNA synthesis and qPCR.

**Additional file 2: Figure S1.** The microclimate regulatory box.

**Additional file 3: Table S2.** Reproductive fitness of gene silenced mosquitoes.

## Abbreviations

cDNA: complementary DNA
Ct-values: threshold crossing values
DNA: deoxyribonucleic acid
dsRNA: double stranded RNA
EIP: extrinsic incubation period
*FN3D1*: fibronectin type III domain-protein 1
*FN3D2*: fibronectin type III domain-protein 2
*FN3D3*: fibronectin type III domain-protein 3
*GPRGR9*: G protein coupled receptor protein 9
HR: hazard ratio
Imd: immune deficiency
IRS: indoor residual spraying
LLINs: long-lasting insecticide-treated nets
*PGRPLC3*: peptidoglycan recognition protein LC3
PBS: phosphate-buffered saline
PCR: polymerase chain reaction
PFA: paraformaldehyde
qrtPCR: quantitative real-time PCR
RNA: ribonucleic acid
TBV: transmission blocking vaccine
