# Supplementary material for: The effect of silencing immunity related genes on longevity in a naturally occurring Anopheles arabiensis mosquito population from southwest Ethiopia
Source: Parasit Vectors. 2019 Apr 16;12:174. doi: 10.1186/s13071-019-3414-y (PMC6469062; doi:10.1186/s13071-019-3414-y)
Supplement: Supplementary file 1 — Additional file 1: Table S1. List of primers used in the dsRNA synthesis and qPCR. [file 13071_2019_3414_MOESM1_ESM.docx]

| Primer Name | Reaction | Sequence |
| --- | --- | --- |
| FN3D1 F | T7, dsRNA preparation | taatacgactcactatagggGATGGACGTGGATCAGCC |
| FN3D1 R | T7, dsRNA preparation | taatacgactcactatagggTGGATCGTCCTCATCACTGT |
| FN3D2 F | T7, dsRNA preparation | taatacgactcactatagggACGGCCGTTTTAAAGTGTCA |
| FN3D2 R | T7, dsRNA preparation | taatacgactcactatagggCCCAGCATTGTTGATGTACAGA |
| FN3D3 F | T7, dsRNA preparation | taatacgactcactatagggAATCATTTCCTTTCGCATTCC |
| FN3D3 R | T7, dsRNA preparation | taatacgactcactatagggACATTGTCCTTGTACCACACCA |
| Gr9 F | T7, dsRNA preparation | taatacgactcactatagggAGCACCCGGCATGCGACATC |
| Gr9 R | T7, dsRNA preparation | taatacgactcactatagggAGCTCAGCTCGTTTTGGCGCA |
| LC3 F | T7, dsRNA preparation | taatacgactcactatagggACGAAATGCATGTATCAGG |
| LC3 R | T7, dsRNA preparation | taatacgactcactatagggTCGTCGGTTTGTGGTGTCGTTC |
| LacZ F | T7, dsRNA preparation | taatacgactcactatagggAGAATCCGACGGGTTGTTACT |
| LacZ R | T7, dsRNA preparation | taatacgactcactatagggCACCACGCTCATCGATAATTT |
| FN3D1 F | qRT-PCR | GGCCGCCGGTTTCATCCACA |
| FN3D1 R | qRT-PCR | TTGCGGTCCAGGTGGTGGGA |
| FN3D2 F | qRT-PCR | GGTGTCGCTGACGGTGACGG |
| FN3D2 R | qRT-PCR | TGCGCCGGAAAGCCGGAAAT |
| FN3D3 F | qRT-PCR | CGTGACGGCCAACGTGACGA |
| FN3D3 R | qRT-PCR | GGTCGCGAACCCACCGACTG |
| Gr9 F | qRT-PCR | CGCTTGTTCTGCTGCATTGT |
| Gr9 R | qRT-PCR | AACGGCCACAGAATGTTTGC |
| LC3 F | qRT-PCR | GCACTGGCAAGGA ACCTAC |
| LC3 R | qRT-PCR | TCGTCGGTTTGTGGTGTCGTTC |
| AgS7F | qRT-PCR | GTGCGCGAGTTGGAGAAGA |
| AgS7R | qRT-PCR | ATCGGTTTGGGCAGAATGC |

**Additional file 1: Table S1. Primer sequences used for dsRNA preparation or qRT-PCR. F, forward; R, reverse (Stathopoulos et al, 2014)**
