# Supplementary figures and images for: The effect of silencing immunity related genes on longevity in a naturally occurring Anopheles arabiensis mosquito population from southwest Ethiopia
Source: Parasit Vectors. 2019 Apr 16;12:174. doi: 10.1186/s13071-019-3414-y (PMC6469062; doi:10.1186/s13071-019-3414-y)

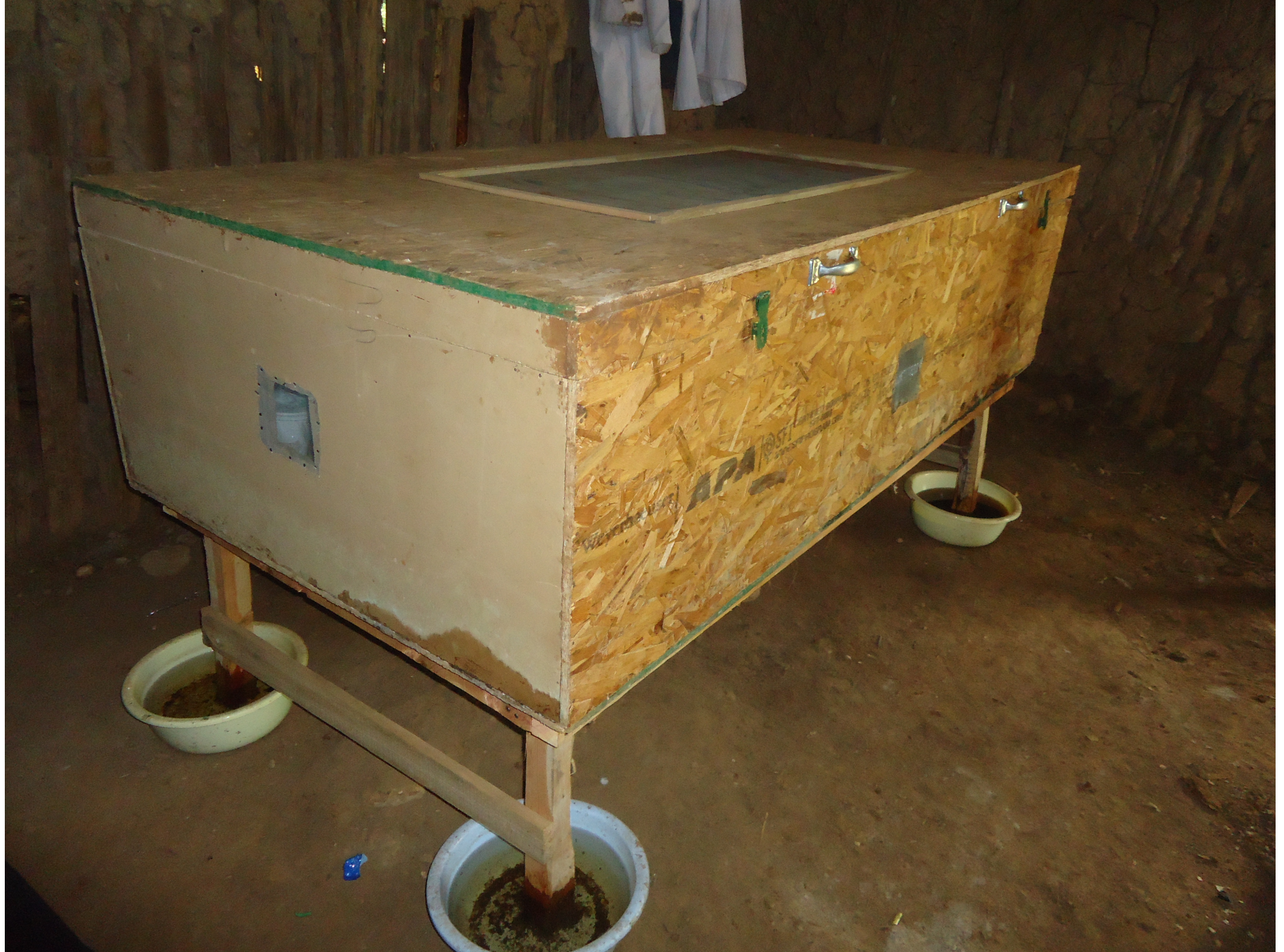

Supplement: Supplementary file 2 — Additional file 2: Figure S1. The microclimate regulatory box. [file 13071_2019_3414_MOESM2_ESM.pdf]
