# Supplementary material for: The effect of silencing immunity related genes on longevity in a naturally occurring Anopheles arabiensis mosquito population from southwest Ethiopia
Source: Parasit Vectors. 2019 Apr 16;12:174. doi: 10.1186/s13071-019-3414-y (PMC6469062; doi:10.1186/s13071-019-3414-y)
Supplement: Supplementary file 3 — Additional file 3: Table S2. Reproductive fitness of gene silenced mosquitoes. [file 13071_2019_3414_MOESM3_ESM.docx]

**Additional file 3: Table S2.** Reproductive fitness of gene silenced mosquitoes. Numbers inside bracket represent number of mosquitoes or eggs tested.

| Gene (kd) | Oviposition (%) | Mean egg/Female | Egg hatchability (%) |
| --- | --- | --- | --- |
| Lacz (14) | 64.3 | 49 (67) | 100% |
| FN3D1 (15) | 60.0 | 42 (62) | 100% |
| FN3D3 (10) | 50.0 | 58 (68) | 80 % |
